# The Brief Symptom Inventory and the Outcome Questionnaire-45 in the Assessment of the Outcome Quality of Mental Health Interventions

**DOI:** 10.1155/2016/7830785

**Published:** 2016-09-08

**Authors:** Aureliano Crameri, Christopher Schuetz, Andreas Andreae, Margit Koemeda, Peter Schulthess, Volker Tschuschke, Agnes von Wyl

**Affiliations:** ^1^Zurich University of Applied Sciences, Zurich, Switzerland; ^2^Integrated Psychiatric Services Winterthur and Zurich Unterland (ipw), Winterthur, Switzerland; ^3^Swiss Charter of Psychotherapy, Stäfa, Switzerland; ^4^University Hospital of Cologne, Cologne, Germany; ^5^Sigmund Freud University, Berlin, Germany

## Abstract

Self-report questionnaires are economical instruments for routine outcome assessment. In this study, the performance of the German version of the Outcome Questionnaire-45 (OQ-45) and the Brief Symptom Inventory (BSI) was evaluated when applied in analysis of the outcome quality of psychiatric and psychotherapeutic interventions. Pre-post data from two inpatient samples (*N* = 5711) and one outpatient sample (*N* = 239) were analyzed. Critical differences (reliable change index) and cut-off points between functional and dysfunctional populations were calculated using the Jacobson and Truax method of calculating clinical significance. Overall, the results indicated that the BSI was more accurate than the OQ-45 in correctly classifying patients as clinical subjects. Nonetheless, even with the BSI, about 25% of inpatients with schizophrenia attained a score at admission below the clinical cut-off. Both questionnaires exhibited the highest sensitivity to psychopathology with patients with personality disorders. When considering the differences in the prescores, both questionnaires showed the same sensitivity to change. The advantage of using these self-report measures is observed primarily in assessing outpatient psychotherapy outcome. In an inpatient setting two main problems—namely, the low response rate and the scarce sensitivity to psychopathology with severely ill patients—limit the usability of self-report questionnaires.

## 1. Introduction

Along with accessibility to services, appropriateness of treatment, and perception of care, outcome is an important quality indicator in mental health care [1]. A widely used approach to assess outcome quality is measuring symptom reduction through rating scales, either clinician-administered or self-reported. Self-report scales are widely used in outcome evaluation of psychotherapies [2]. Their standardized form allows easy administration by clinicians without additional training and also guarantees a high level of reliability. On the other hand, for patients with a high degree of psychopathology, clinician-administered scales are preferred [3], for with these scales, the psychological status of each patient can be assessed independently of the person's capability or willingness to accurately describe their relevant symptoms and behaviors. However, achieving good rating quality requires clinician training in the application of these instruments plus subsequent assessment of interexaminer reliability. Given that in general psychiatric hospitals it has been proven to be quite difficult to routinely involve clinicians in training courses, the question arises as to whether clinician-rated assessments can be substituted by similar scales filled out by psychotherapy patients themselves (self-report) [4].

For general assessment of mental health symptoms, one of the frequently suggested multidimensional self-report instruments is the Symptom Checklist (SCL-90R) or its short form, the Brief Symptom Inventory (BSI) [5–7]. However, based on the number of published studies implementing these questionnaires, it can be asserted that in the field of mental health they have been used predominantly with outpatients and far less frequently with inpatients or, more generally, with patients with severe mental illness [8–10]. Nevertheless, these instruments seem promising in assessing the mental health status of the latter patient group, because they also contain specific scales for measuring psychotic and schizotypal symptoms, such as the Psychoticism or the Paranoid Ideation scale. However, the validity of these scales has not as yet been unequivocally confirmed. Wood [11] found no evidence that patients with schizophrenia score higher on the Psychoticism scale than patients without schizophrenia. Johnson et al. [12] found no differences on the Paranoid Ideation and Psychoticism scales between patients diagnosed with or without schizophrenia. In contrast, Preston and Harrison [13] demonstrated in a sample of 69 patients presenting their first psychotic episode that responses on the BSI items could discriminate between patients with weak and with marked positive symptoms.

Another instrument recommended for assessment of individuals seeking mental health treatment is the Outcome Questionnaire-45 (OQ-45) [14–16]. It is a newer, self-report outcome measure created primarily for psychotherapy patients, which has already been used in psychiatric inpatient care [17, 18].

In the context of quality assurance programs, quantitative data collected via mental health questionnaires find their application in the analysis of pre-post differences, which reflect the intraindividual changes attained during the treatment. One widely used analysis method is the calculation of clinical significance proposed by Jacobson and Truax [19]. The aim of the method is to identify which patients move outside the range of the dysfunctional population and consequently attain “normal” functioning. Patients who improve significantly and cross a well-defined cut-off score between dysfunctional and functional distributions are classified as “remitted.” However, patients can only be consistently classified in this way if the self-report measurement at the beginning of the treatment correctly identifies them as dysfunctional.

The objective of this study was to evaluate* the German versions of the OQ-45 and the BSI when applied in the analysis of outcome quality of inpatient and outpatient treatments*. The analyses focused on three aspects of usability:

1. *Response Rate*. How many patients fill out the questionnaires? To produce a representative outcome estimation, the majority of the patients should be able to fill out the self-report forms. A high rate of nonresponse increases the risk of bias in the results.
2. *Sensitivity to Psychopathology*. Applying the cut-off scores calculated with the Jacobson and Truax method, what percentages of patients are correctly classified by the self-report measures at admission as belonging to the dysfunctional population?
3. *Sensitivity to Change*. Do the two questionnaires have the same sensitivity to change? The equivalence of the two measures is not obvious, since compared to the BSI, the OQ-45 contains fewer symptom-specific items and focuses partially on social functioning (e.g., work), which requires more time for change to occur than the time required to see change in acute symptoms.

## 2. Methods

### 2.1. Samples

Three different samples, two inpatient samples and one outpatient sample, were used for this study. Both inpatient samples were treated at an inpatient clinic in Switzerland, the Integrated Psychiatric Services of Winterthur, and Zurich Unterland (ipw).

At the ipw, the OQ-45 was implemented as a self-report measure of outcome quality from 2008 to 2010; it was replaced by the BSI starting in 2012. For samples 1 and 2, inpatient treatments from 2008 to 2009 (OQ-45) and from 2012 and 2013 (BSI) were selected from the database using the following inclusion criteria:

- A principal diagnosis belonging to one of these major groups: F1 (substance abuse), F2 (schizophrenia or other psychotic disorders), F3 (mood disorders), F4 (anxiety or stress related disorders), and F6 (personality disorders).
- Age of at least 18 years.
- Hospitalization of at least 7 days.

Sample 3 was recruited in a project on outpatients promoted by the Swiss Charta for Psychotherapy. The aim of this nonrandomized field study is to investigate various process-outcome aspects of outpatient treatments carried out with different experiential or psychodynamic therapy methods [26–28]. Cases from this sample were selected using the following criteria:

- At least one Axis I disorder according to the criteria of DSM-IV.
- Age of at least 18 years.
- Minimum of 10 treatment sessions.

Both studies above were carried out in accordance with the ethical principles of the Declaration of Helsinki. For the Swiss Charta project, a research application was submitted to the ethics committee of each of the Swiss cantons in which the projects were carried out; all of the applications were approved. All patients gave informed written consent for their participation in the study. Data from the ipw were collected within an internal quality measurement system for which, according to Swiss federal and cantonal law, no ethical approval was required. For this reason, the present study was granted an exemption from the requirement for ethics approval by the Cantonal Ethics Committee of Zurich (Waiver number 09-2016). Participating patients at the ipw gave informed verbal consent for the use of the data.

Table 1 presents the characteristics of the three samples. In the inpatient samples 1 and 2 the most frequent substance-related disorder (F1) was associated with alcohol consumption (71.5% of the patients with an F1 diagnosis in sample 1 and 56.2% in sample 2, resp.). In the F2 group paranoid schizophrenia was prominent (61.8% and 59.3%, resp., of the F2 group). Most of the patients with a mood disorder had either a depressive episode (53.2% and 51.0% within the F3 group) or a recurrent depressive disorder (40.3% and 45.4% within the F3 group). The most frequently diagnosed personality disorder was of the Borderline type (64.6% and 53.6% within the F6 group). In the outpatient setting, 69.2% of diagnosed mood disorders were major depressions. The most frequent anxiety disorders in this setting were social phobia, panic disorder/agoraphobia, and generalized anxiety disorder.

### 2.2. Measures

The following self-report and clinician-administered instruments were used for this analysis:

- *Basic Documentation*. Important clinical information and sociodemographic characteristics were recorded on a form. These data were collected by the treating psychiatrists (for inpatients) and the treating psychotherapists (for outpatients).
- *ICD-10*. Diagnoses based on this system were available for the inpatient samples and had been assigned by the treating psychiatrists.
- *Structured Clinical Interviews for DSM-IV (SCID-I/-II) [29]*. These interviews, which were available only for sample 3, served in assessing Axis I and II disorders (clinical syndromes and personality disorders) according to the DSM-IV criteria. Trained psychologists (not involved in treatment of the patients) were engaged to carry out the two clinical interviews.
- *Clinical Global Impressions (CGI) [30]*. This instrument gathers the clinician's view of the severity of psychopathology (CGI-S) and of the improvements from the initiation point of the treatment (CGI-I). The two aspects are each rated on a 7-point scale. Ratings with this instrument were available only for the inpatient samples and were provided by the treating psychiatrists.
- *Global Assessment of Functioning (GAF) [31]*. On this scale psychological, social, and occupational functioning are rated on a hypothetical continuum from severe mental illness (0) to mental health (100).
- *OQ-45*. The questionnaire consists of 45 items grouped in the three scales Symptom Distress (SD), Interpersonal Relations (IR), and Social Role (SR), which add up to a total score (OQ Total Score). The scale structure of the original version is supported by confirmatory factor analysis [14]. The single scales in the German version exhibit good internal consistency [20].
- *BSI*. This questionnaire is the short version of the SCL-90R [6]. The 53 items assess nine primary symptom dimensions: Somatization (SOM), Obsessive-Compulsiveness (OBS), Interpersonal Sensitivity (INS), Depression (DEP), Anxiety (ANX), Hostility (HOS), Phobic Anxiety (PHOB), Paranoid Ideation (PAR), and Psychoticism (PSY). Additional global indices of distress can be obtained, for example, the General Symptom Index (GSI), which is the mean score of all items. In the analysis carried out by Derogatis and Melisaratos [5] seven of the primary scales showed a clear convergence with their counterparts among the MMPI scales. All scales in the German version exhibit satisfactory test-retest reliability [22].

### 2.3. Statistical Analysis

#### 2.3.1. Missing Data

**Figure 1:**
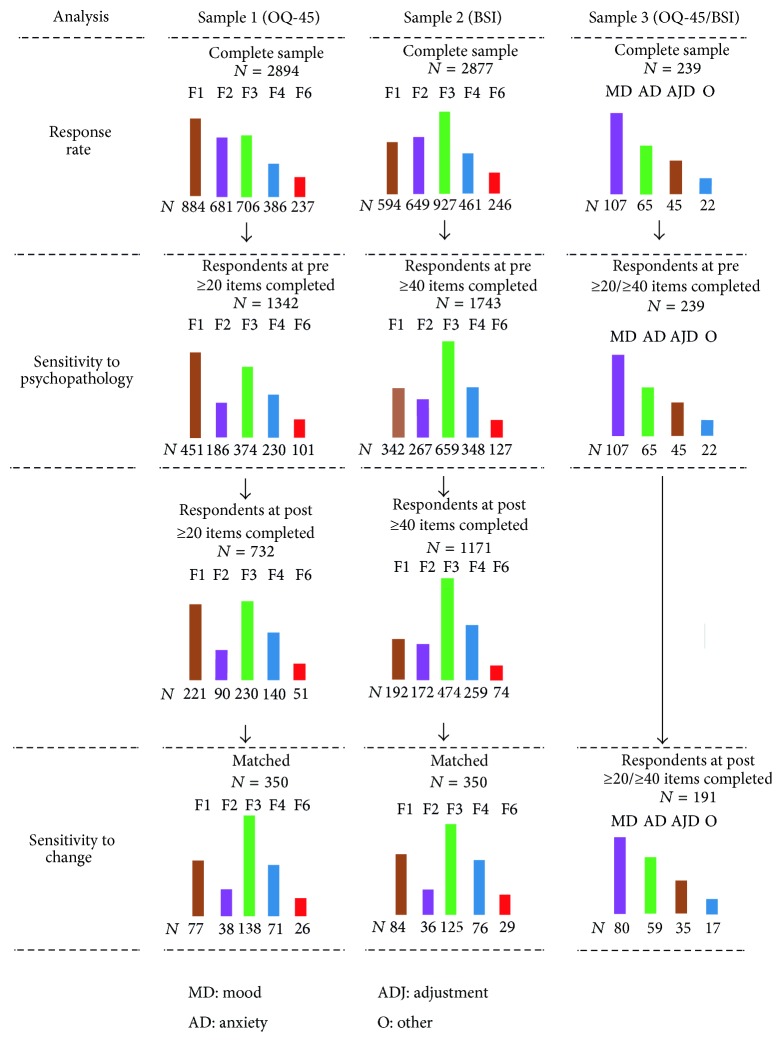
Number of cases included in the different analyses.

Nonresponse is a common problem in surveys and can occur for single items or for the entire examination. Analyzing only data of patients that responded to every item on the self-report measures at admission as well as at discharge would lead to a substantial loss of information, especially for the analyses for the inpatient setting. To include as many cases as possible, we defined different inclusion criteria for each analysis (Figure 1). First, we defined the minimal number of answered items to calculate scale scores. In a previous unpublished study we tested (through simulations) the impact of incomplete item values in the calculation of scores for the OQ-45 scales. Our results showed that if, for the three scales, namely, SD, IR, and SR, at least 8, 6, and 6 items, respectively, had a valid value, then the generated scale scores preserved a correlation of *r* > 0.90 with the corresponding scores based on completely observed values. In this study, we considered as evaluable only data records that fulfilled the rule mentioned above. Concerning the BSI, we analyzed only returned questionnaires with at least 40 valid item values [32].

We calculated the response rate on the basis of the number of returned questionnaires that fulfilled the above-mentioned criteria of completeness. Patients who completed a sufficient number of items at admission were included in the analysis of sensitivity to psychopathology. Patients who additionally did the same at discharge were included in the analysis of sensitivity to change.

#### 2.3.2. Parameters of Clinical Significance

For the analysis of intraindividual changes we applied Jacobson and Truax's method of calculating clinical significance [19], with which the proportions of improved and recovered cases can be calculated [2]. To determine these proportions, two parameters are needed: (1) a critical difference *D*, which allows identification of individual pre-post differences that are sufficiently large to be considered statistically significant (reliable change) and (2) a cut-off *C*, which distinguishes scores that are a variation of normal functioning from scores indicating a psychopathological state. Therefore, patients exhibiting a reduction of at least *D* points from their initial impairment score are considered “improved.” If their score additionally falls below the cut-off *C*, then they are considered “remitted.”

The critical difference, for example, at a confidence level of 95%, is obtained by using the standard deviation and the reliability coefficients of the measure:

(1) $$\begin{array}{c} D_{95\%}=1.96\cdot {\text{SD}}_{\text{patient}}\cdot \sqrt{2\left(1-r\right)}, \end{array}$$

whereas the cut-off is calculated as a weighted midpoint between the means of a patient and a nonpatient population as follows:

(2) $$\begin{array}{c} C=\frac{{\text{SD}}_{\text{patient}}\cdot M_{\text{nonpatient}}+{\text{SD}}_{\text{nonpatient}}\cdot M_{\text{patient}}}{{\text{SD}}_{\text{patient}}+{\text{SD}}_{\text{nonpatient}}}. \end{array}$$

We additionally compared our parameter values with those published in other studies on the basis of samples from Germany.

#### 2.3.3. Outcome Comparisons

To compare sensitivity to change, measured with two different questionnaires within three different samples, the following analysis techniques were used: propensity score matching and linear mixed modeling. Given that within the inpatient setting OQ-45 and BSI data were collected in two different samples, we matched sample 1 and sample 2 using the propensity score technique proposed by Rosenbaum and Rubin [33]. It is primarily used in nonrandomized studies in order to build equivalent samples for causal inference, but it can also be applied to compare different survey samples [34]. In our analysis treatment modalities were defined as the two different questionnaires presented to the patient (BSI versus OQ-45), and outcome was defined as the scale scores measured at intake and at discharge and then converted either in categories of clinical significance or in *z* values. As a matching algorithm we applied the nearest neighbor matching with a logistic regression-based propensity score [35]. The following covariates were used in the regression equation: sex, age, marital status, educational level, GAF and CGI, respectively, at intake and at discharge, principal diagnosis, compulsory treatment order, duration of treatment, and type of discharge. Matches were formed on a 1 : 1 ratio within a caliper size of 0.1 standard deviations of the propensity score. The adequacy of the matching procedure was checked using graphical visualizations of the propensity score distribution; the quality of the balance in the matched groups was examined through the standardized difference of means of each covariate [36, 37].

Besides the comparison of the proportions of cases in the principal categories of clinical significance classified by the two instruments, we also analyzed the pre-post differences using the linear mixed model; for this, the OQ-45 and BSI scores were *z*-transformed.

## 3. Results

### 3.1. Response Rate

The completeness of the returned questionnaires varied between the two measures. The highest number of unanswered items was found for the OQ-45 questionnaires returned by the inpatients. Questions on intimate relationships but also work-related questions were the most affected by nonresponse in sample 1. Item number 7 (“I feel unhappy in my marriage/significant relationship”) was left out in 24% of the cases. A work-related item such as number 12 (“I find my work/school satisfying”) had 8% missing values. Overall, the impact of incomplete responses in sample 1 was high: of the 1342 questionnaires evaluable at intake only 588 (43.8%) had been completely filled out.

Nonresponse on the BSI questionnaires returned by inpatients was not related to the content but instead to the position of the items. Items 1 to 10 had on average 1.5% missing values; items above number 40 reached missingness from 4% to 7.5%. Of the 1743 evaluable BSI forms at intake only 1282 (73.6%) were complete.

In the outpatient sample the incompleteness of the questionnaires was low. For the OQ-45, besides a 7% of nonresponse on item number 7, missingness varied from 0% to 3.5%. On the BSI questionnaires, missingness on the single items was always under 1%.

In the inpatient setting the response rate at both pre- and postmeasurement was about 25% for the OQ-45 in sample 1 and 40% for the BSI in sample 2. This difference has to be viewed as improvement in data monitoring over the course of the years and cannot be considered to be an indicator for better acceptance of the BSI over the OQ-45.

**Figure 2:**
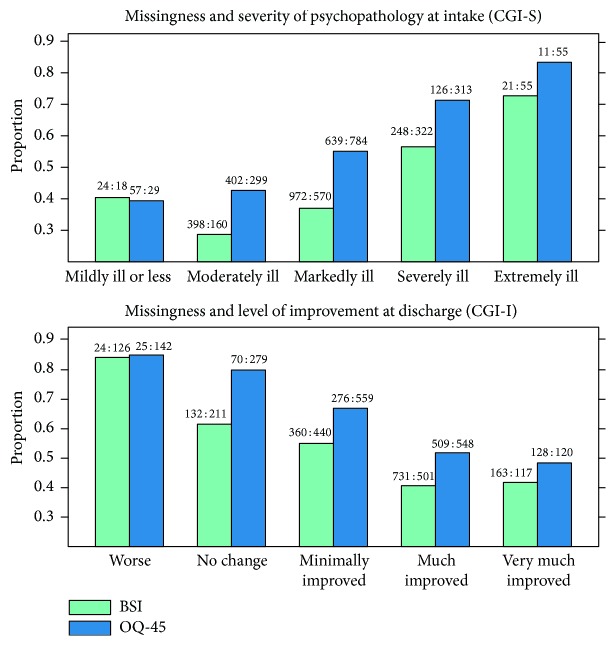
Relationship between missingness (proportion of nonresponse) and CGI ratings. The ratio between the number of respondents and the number of nonrespondents is noted on top of the bars (data from samples 1 and 2).

The response rate was dependent on the severity of psychopathology at admission and of the amount of improvement at discharge in both inpatient samples (Figure 2). Moreover, patients with schizophrenia showed the lowest response rate, with the BSI, 41% at intake and 45% at discharge. The highest rate was registered among patients having an F4 diagnosis, with 75% at intake and 65% at discharge, respectively.

In the outpatient sample the response rate at the end of the therapy did not correlate significantly with Axis I or Axis II diagnoses.

### 3.2. Cut-Off and Critical Difference

Table 2 shows the descriptive statistics of the scales based on data collected at the beginning of the treatment in the three samples.  Table 3 reports comparative statistics from German sample 3.

The cut-off and the critical difference for the OQ Total Score were calculated using the data of the respondents from sample 1 at intake (*N* = 1342, *M*_patient_ = 78.4, SD_patient_ = 28.9, and Chronbach's *α* = 0.95) and data published by Lambert et al. [20] on a nonclinical sample (*N* = 232, *M*_nonpatient_ = 46.2, and SD_nonpatient_ = 18.5). Applying (1) and (2) led to *C* = 59 and *D*_95%_ = 18. Therefore, a patient can be classified as remitted if the following two conditions are fulfilled: (a) his OQ Total Score has decreased by at least 18 points and (b) has reached a value below 59 at the end of the treatment. If only (b) is met, then the patient is classified as improved. Both parameter values are similar to those published in Puschner et al. [18].

The calculation of the parameters for the GSI scale was based on data of the respondents from sample 2 (*N* = 1743, *M*_patient_ = 1.46, SD_patient_ = 0.79, and Chronbach's *α* = 0.97) and the data published in Franke [22] on a nonclinical sample (*N* = 600, *M*_nonpatient_ = 0.31, and SD_nonpatient_ = 0.23). The corresponding parameters of clinical significance were *C* = 0.57 and *D*_95%_ = 0.38. Schmitz et al. [24] obtained a similar cut-off value based on a sample of participants with severe impairments in which inpatient treatment was indicated. Lutz et al. [25] proposed a critical difference calculated with the standard deviation of the functional sample, which is consequently narrower than our value based on a commonly larger standard deviation from a clinical sample.

### 3.3. Sensitivity to Psychopathology

**Figure 3:**
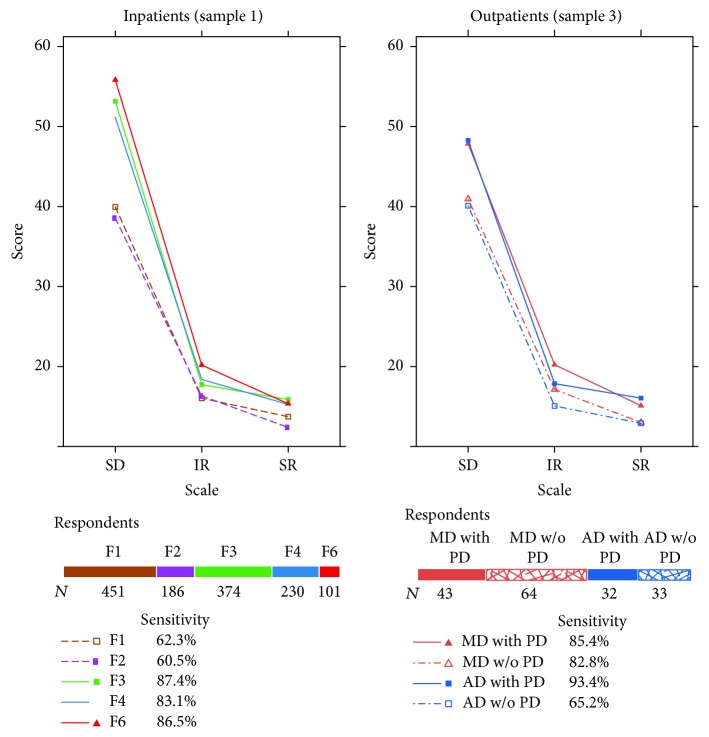
OQ-45 profiles of the respondents at intake. For sample 3 only data of patients with a principal diagnosis of mood (MD) or anxiety disorder (AD) grouped according to the presence or absence of a personality disorder (PD) are represented. Percentages represent the sensitivity to psychopathology according to the OQ Total Score.

**Figure 4:**
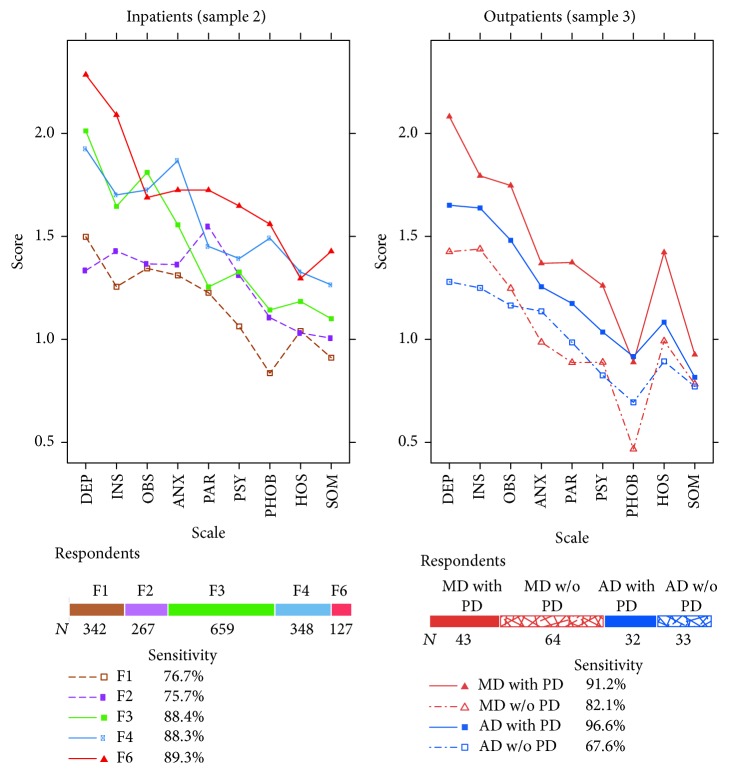
BSI profiles of the respondents at intake. For sample 3 only data of patients with a principal diagnosis of mood (MD) or anxiety disorder (AD) grouped according to the presence or absence of a personality disorder (PD) are represented. Percentages represent the sensitivity to psychopathology according to the GSI scale.

Figures 3 and 4 report the sensitivity of the global scales OQ Total Score and GSI.  Figure 3 shows the profiles of different diagnosis groups on the OQ-45 scales. More than 80% of the inpatients with diagnoses F3, F4, and F6 had an OQ Total Score of at least 59 points and were therefore classified as clinical cases (caseness). Fewer than 70% of inpatients with a diagnosis F1 or F2 scored above the clinical cut-off. Considering the most important clinical disorders in an outpatient setting, that is, affective and anxiety disorders, fewer than 70% of the patients with an anxiety disorder but without a comorbid personality disorder (PD) were correctly classified as clinical cases by OQ Total Score. In contrast, sensitivity of more than 80% was attained among patients with an affective disorder, independently of additional comorbidity on the Axis II. Since the SD scale contributes the most to the Total Score scale, a high correlation of *r* = 0.96 between the two scales was present. The other two scales, that is, IR and SR, which have a minor importance in forming the Total Score, also did not vary substantially across the different type of disorders.

In contrast to the OQ-45, the BSI produced profiles with more distinctive differences between the diagnosis groups (Figure 4). Except for the patients with schizophrenia (F2), the Depression scale was the scale with the highest score for the different diagnosis groups. The largest mean on this scale was exhibited by patients with a personality disorder (F6) and not, as theoretically expected, by patients with an affective disorder (F3). Patients with schizophrenia attained the highest score on the Paranoid Ideation scale, which would be in accordance with the diagnostic criteria for this psychiatric group. However, their mean score was outperformed by that of patients with an F6 diagnosis. Overall, the highest Symptom Distress as measured by the BSI was observed on average among patients with a personality disorder in both an inpatient and an outpatient setting.

In the samples of inpatients and outpatients, the BSI with its GSI scale exhibited a higher sensitivity to psychopathology than the OQ-45 with its Total Score scale. However, the same diagnosis groups with a low average OQ Total Score, that is, F2 and F1 among the inpatients and anxiety disorders without PD among the outpatients, achieved a GSI score that was on average also lower than that of other diagnosis groups. About 25% of the respondents with an F2 diagnosis were not correctly classified as clinical cases. The caseness determined by the GSI scale was not associated with the CGI or GAF score (logistic regression: *χ* ^2^(2) = 0.56, *p* = 0.76). Therefore, the substantial misclassification of patients with schizophrenia cannot be considered to be a consequence of low severity of mental illness or high psychosocial functioning of the sample analyzed.

For both instruments one would actually expect to find higher sensitivity to psychopathology with inpatients than with outpatients, but the percentages in Figures 3 and 4 do not support this expectation. The statistics in Table 2 show that respondents with 0 points on one of the questionnaires were found among the inpatients but not among the outpatients and that the 5th and the 10th percentiles in the inpatient samples were lower than the corresponding values from the outpatients. The lowest 5th percentiles of the global questionnaire scores were found for the GSI among the inpatients with substance abuse (0.15) and inpatients with schizophrenic disorders (0.11).

### 3.4. Sensitivity to Change

**Figure 5:**
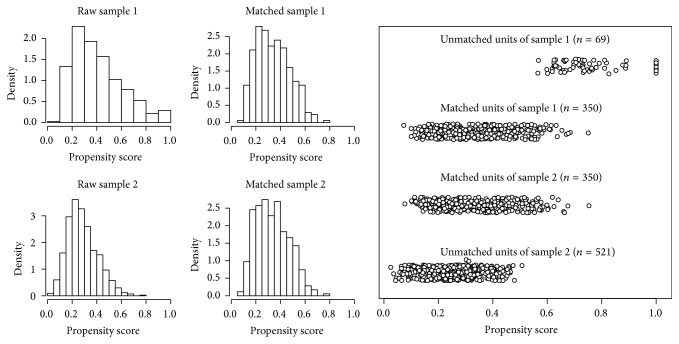
Distributions of the propensity scores of the inpatient respondents with complete covariates values. Histograms on the left show the distributions before (raw) and after (matched) the matching. The plot on the right shows the differences between matched and unmatched cases.

For the inpatient setting, we based our analysis of sensitivity to change on matched samples. The histograms in Figure 5 show that samples 1 and 2 were already quite similar before the matching, since they exhibited an extensive overlap of their propensity score distributions but with some density differences, however. From the smaller sample, that is, sample 1, a total of 419 patients with complete scales and covariates values were available for the matching. Of these, 350 cases could successfully be matched on a 1 : 1 ratio with cases from the larger sample 2 (the absolute standardized difference of means was smaller than 0.1 for each covariate).

**Figure 6:**
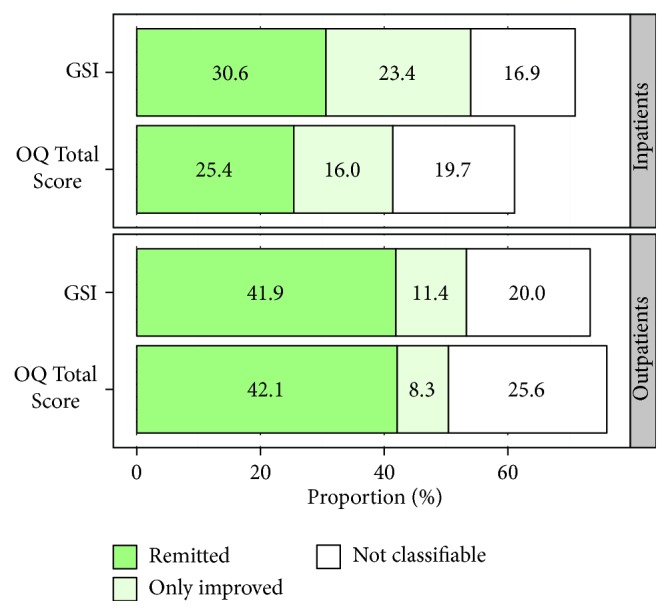
Results of the analysis of the clinical significance. Analysis of inpatient data are based on matched samples: *n*_1_ = *n*_2_ = 350. Outpatient sample: *n*_3_ = 191. The category “not classifiable” refers to cases misclassified as nonclinical by the self-report measure.

Figure 6 shows the results of the clinical significance analysis. In the outpatient sample, the proportions of improved and remitted cases classified by the two measures were quite similar. In the inpatient samples, the GSI scale identified 54% improved cases compared to the 41.4% identified by the OQ Total Score. Although this difference is significant [*χ* ^2^(7) = 58.5, *p* < 0.001], it does not necessarily attest a superior sensitivity to change to the BSI. This is because the GSI scale produces higher prescores than the OQ Total Score, and, therefore, patients have a higher probability of lowering their score on the GSI than on the OQ total scale during their treatment.

**Figure 7:**
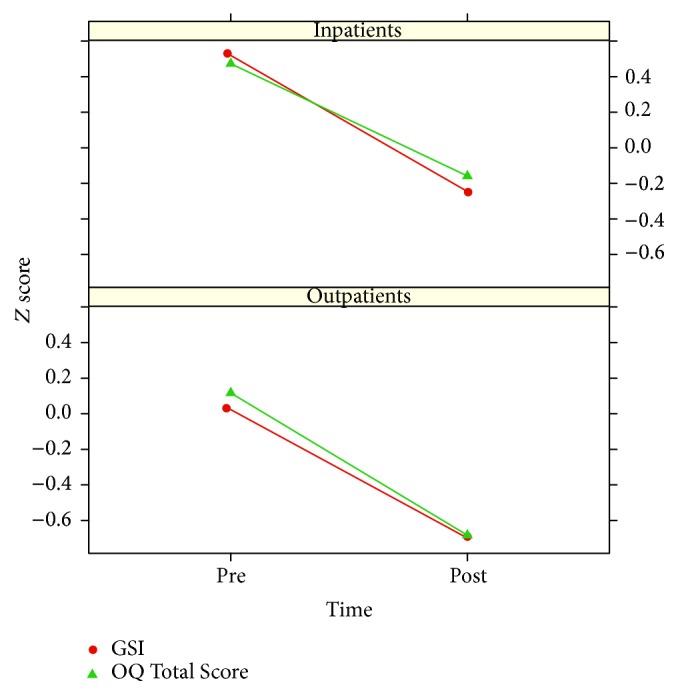
Average pre-post changes in *z* scores. Analysis of inpatient data are based on matched samples: *n*_1_ = *n*_2_ = 350. Outpatient sample: *n*_3_ = 191.

To balance out this difference, the scores of the two questionnaires were *z*-transformed and analyzed with a linear mixed model.  Figure 7 shows the estimated fixed effects; they indicate that the two questionnaires recorded the same amount of change between the pre- and the postmeasurement.

## 4. Discussion

This study examined the applicability of the OQ-45 and the BSI for assessing the outcome quality of inpatient and outpatient treatments. Both of these self-report measures can be easily administered by a wide range of service professionals and take about 10 minutes to be filled out. Normative data and analysis results concerning their psychometric properties are available [38].

However, our analyses pointed out the following critical aspects of the performance of the two questionnaires, which have often been neglected in the literature: (1) the number of missing values that emerges in the data collection, (2) the diagnostic value of the scale profiles, and (3) the robustness of the clinical significance algorithm.

(*1) Missing Data*. Since Rubin's [39] seminal paper on inference with missing data, there has been a growing awareness of this problem in the scientific community. In evaluation studies, the probability of nonresponse is often correlated with the attained outcome. Our results clearly demonstrate this relationship. Respondents and nonrespondents are different in two clinically crucial aspects: nonrespondents have higher severity of mental illness and show less improvement after the treatment than respondents. Missingness is therefore a source of bias when assessing the effectiveness of a treatment, and nowadays guidelines concerning the statistical analysis of incomplete data are available [40, 41]. Different authors have pointed out that missingness as low as 10%, if not treated adequately in the statistical analysis, can lead to bias [42, 43]. In a previous analysis we were able to successfully apply multiple imputation to the outcome data of sample 3, because, first, the missing rate in the sample was relatively low (20%) and, second, the progress of the patients was additionally monitored through repeated measurements during the treatment, which generally improve the predictive power of the imputation model [27]. In contrast, with samples 1 and 2 the application of multiple imputation proved to be ineffective. On the one hand, nonresponse in these samples exceeded 50%, and according to Rubin [44] multiple imputation was conceived to deal with a typical fraction of missing information of 30% or less. On the other hand, the drastic reduction of the data collection to only pre-post measurements, in order to minimize administrative expense, makes it hard to obtain robust estimations of the missing data through imputation models. All in all, the high proportion of missing data discourages the use of self-report measures with the patients with severe impairments usually found in a psychiatric hospital setting in favor of clinician-administered measures.

(*2) Diagnostic Value of the Profiles*. Questionnaires like OQ-45 and BSI have a relatively large number of items, so that different reliable Likert scales may be formed that allow the creation of a person's profile. Can these profiles be used, for instance, to facilitate the formulation of a psychiatric diagnosis? Our results do not support the use of these questionnaires as screening instruments to facilitate the assessment of ICD-10 diagnoses. In the construction of the OQ-45 this was never an intended purpose [45], but the nine primary symptom dimensions of the BSI suggest a possible application for screening purposes. In our sample, inpatients with personality disorders attained on average higher scores than inpatients in other diagnosis groups on six of the nine BSI scales. These patients had a higher mean score on the Depression scale than patients with an affective disorder and a higher mean score on the Paranoid Ideation scale than patients with schizophrenia. Poor diagnostic efficacy of the nine scales in assessing DSM-IV symptom disorders was also reported by Pedereen and Karterud [46]. Two different approaches have been suggested for dealing with the low discriminant validity of the BSI scales. The first is to consider the questionnaire through its GSI score as more appropriate for measuring the overall degree of psychological distress instead of the precise nature of the psychopathology [38]. From this optic, the Outcome Questionnaire with 45 items in its full version or 30 items in its short version would seem to be a more time-effective choice than BSI when measuring general level of psychological distress in a less time-consuming way. The second approach consists in improving the factorial structure of the questionnaire. To this purpose, different authors have used the bifactor structural model in recent years. This model is used to build a general distress factor and more specific components of psychopathology. Thomas [47] demonstrated that a bifactor model of the BSI items can achieve higher accuracy than an oblique simple structure in diagnosing some disorders, such as depression or generalized anxiety disorder. Brodbeck et al. [48] also proposed a bifactor solution that correlates with DSM-IV diagnoses, especially depressive disorders, anxiety disorders, and personality disorders. One of their results in line with ours is that patients with personality disorders are characterized by a high level of general distress. Overall, it seems that improving the factor structure of the BSI can lead to improved sensitivity in identifying depressive or anxiety disorders, but we doubt that it can do the same with patients with substance abuse or acute psychotic disorders. A nonnegligible part of these patients tends to score low, and their profiles resemble those of healthy persons or remitted patients. These results support the hypothesis that patients with these disorders are inclined to underestimate their own emotional and behavioral difficulties. As pointed out by Burlingame et al. [8] the disadvantages of using self-report measures with inpatients with severe and persistent mental illness “include an insufficient clinical picture as a result of the dependence on patients' ability to accurately describe their condition, which at times is doubtful because of denial, minimization of symptoms, or responder bias” (p. 448).

(*3) Robustness in the Outcome Evaluation*. Clinical significance, as originally proposed by Jacobson and Truax [19], is considered, beside pre-post effect sizes, to be a gold standard as a performance indicator in routine outcome monitoring [49]. This approach encompasses two steps: first, identifying the subsample of patients that reached a reliable change and then determining which among them moved outside the range of the dysfunctional population. This method presupposes a valid and consistent distinction between functionality and dysfunctionality already* at the onset of the treatment*. Therefore, the percentage of cases that can be adequately categorized depends on the sensitivity of the instrument. For self-report questionnaires measuring important mental health problems, the expected sensitivity is of at least 80% [50, 51]. Our results reveal a heterogeneous picture in this regard. For inpatients with an F3, F4, or F6 diagnosis the sensitivity to psychopathology of both instruments exceeds 80%. However, the questionnaires exhibited low values with inpatients with a principal diagnosis of substance abuse or schizophrenia. Especially within the latter group, which represents an important diagnosis group in an inpatient setting, about 25% of the respondents were misclassified as nonclinical subjects according to the GSI score. The patients with schizophrenia analyzed in this study had a mean GAF score of 32 points at admission and were hospitalized on average 36 days in a primary care hospital. Thus, the substantial misclassification cannot be explained by a lack of mental illness but instead by limitations, either of the measures used in assessing psychopathology or of the methodology used in assessing caseness. Analyses based on outpatients have reported sensitivity of above 90% for the OQ total scale [52] as well as the GSI scale [46]. Sensitivity values of at least 80% based on inpatient data were reported by Moessner et al. [53] for both global scales using data from inpatients with an F3, F4, F5, or F6 diagnosis and by Timman et al. [54] for the OQ Total Score using an inpatient sample with personality disorders. Nevertheless, our analyses do not support a generalization of this high accuracy in detecting caseness among inpatients with an F1 or F2 diagnosis. A lack of research on the generalizability of the OQ-45 was confirmed by Lambert and Hawkins [55], who admitted that much of the research “demonstrating the use of the OQ-45 has been conducted with young, educated patients” (p. 496).

Concerning the limitations of our methodology in assessing clinical significance, we see the following possibilities for improvement: (1)* construct psychological functioning scales using a bifactor model*. For both questionnaires, there are various published results demonstrating that this kind of factor analysis is able to improve the fit of the scale structure to the data [47, 48, 56]; (2)* determine the cut-off scores with the receiver operating characteristic (ROC) curve*. This method can provide more accurate cut-off scores than the weighted midpoints calculated by the Jacobson and Truax method, especially when data are not normally distributed [57]. The procedure requires raw data from both a healthy and a patient sample; however, (3)* avoid the necessity of cut-off scores between functionality and dysfunctionality by using the percentage of improvement approach*. A different outcome evaluation that could be applied as a complement to the Jacobson and Truax method consists in analysis of the relative change from the baseline severity [58]. A reduction of at least 50% of the initial symptom level can be considered as response to treatment, whereas a reduction of at least 75% is necessary to rate the outcome as remission [59].

Concerning the comparison of the two outcome instruments, the overall better accuracy of BSI in detecting clinical cases that emerged in our analyses means that patients' prescores are on average lower with the OQ-45 than with the BSI questionnaire. Patients with low scores already at the beginning obviously have on average a minor probability of further lowering their scores during the treatment, independently of the quality of the treatment. Consequently, this disparity between the two measures leads to a higher sensitivity to change for the BSI.

## 5. Conclusion

On the whole, the comparison of the two questionnaires, BSI and OQ-45, as instruments to be used for routine outcome assessment leads to the following statements:

- In an* inpatient setting* both questionnaires have basically the same* sensitivity to change*. However, the OQ-45 has a lower* sensitivity to psychopathology* than the BSI, a characteristic that also has an impact on sensitivity to change. Another drawback affecting the OQ-45 is that inpatients tend to leave out the questions on intimate relationships or work. Unfortunately, in the inpatient setting, the nonresponse rate with both self-report measures is higher than 30%, leading to potentially nonrepresentative results.
- In an* outpatient setting*, the superiority of the BSI in* both types of sensitivity* is minimal. Therefore, OQ-45 can be considered as an equivalent alternative to the BSI in routine outcome monitoring, with the advantage that it is less time-consuming.
- Due to the limited sensitivity to psychopathology in* both treatment settings,* it is not advisable to base clinical assessment on data collected only with these self-report questionnaires; they should be complemented with clinician-completed ratings.

## Figures and Tables

**Table 1:** Descriptive statistics of the analyzed samples.

	Sample 1 (*N*_1_ = 2894)	Sample 2 (*N*_2_ = 2877)	Sample 3 (*N*_3_ = 239)
Sex			
Female	1424 (49.2%)	1532 (53.2%)	162 (67.8%)
Male	1470 (50.8%)	1345 (46.8%)	77 (32.2%)
Education			
Low	1005 (34.7%)	895 (31.1%)	24 (10.0%)
Middle	1461 (50.5%)	1488 (51.7%)	86 (36.0%)
High	428 (14.8%)	494 (17.2%)	129 (54.0%)
Income			
Salary	729 (25.2%)	687 (23.9%)	185 (77.4%)
Sickness/disability benefit	1060 (36.6%)	1147 (39.9%)	23 (9.6%)
Social welfare payments	425 (14.7%)	373 (13.0%)	3 (1.3%)
Old age insurance	232 (8.0%)	292 (10.1%)	7 (2.9%)
Other	448 (15.5%)	378 (13.1%)	21 (8.8%)
GAF	47.0 (17.2)	38.5 (14.3)	60.5 (13.1)
Principal diagnosis (ICD-10/DSM-IV)	F1: 884 (30.6%)	F1: 594 (20.6%)	Mood: 107 (44.8%)
	F2: 681 (23.5%)	F2: 649 (22.6%)	Anxiety: 65 (27.2%)
	F3: 706 (24.4%)	F3: 927 (32.2%)	Adjustment: 45 (18.8%)
	F4: 386 (13.3%)	F4: 461 (16.0%)	Other: 22 (9.2%)
	F6: 237 (8.2%)	F6: 246 (8.6%)	
Duration of treatment	36.7 days (38.1)	36.0 days (31.7)	41.3 sessions (34.2)
Type of discharge			
Mutual consent	2327 (80.4%)	2489 (86.5%)	180 (75.3%)
Decided by the patient	221 (7.6%)	117 (4.1%)	46 (19.2%)
Decided by the treating person	215 (7.4%)	181 (6.3%)	11 (4.6%)
Other	131 (4.6%)	90 (3.1%)	2 (0.1%)

**Table 2:** Descriptive statistics and reliability (Chronbach's alpha) of the global scales.

	OQ Total Score		GSI	
	Inpatients (*n*_1_ = 1342)	Outpatients (*n*_3_ = 239)	Inpatients (*n*_2_ = 1743)	Outpatients (*n*_3_ = 239)
M (SD)	78.4 (28.9)	72.0 (19.4)	1.46 (0.79)	1.06 (0.53)
Min	0	33	0	0.26
Percentiles				
5	31	40	0.25	0.34
10	38	47	0.41	0.45
25	58	60	0.85	0.66
50	79	71	1.40	0.97
75	99	85	2.02	1.39
90	117	95	2.56	1.76
95	126	104	2.85	2.02
Max	159	143	3.79	2.78
*α* ^*∗*^	0.95	0.91	0.97	0.95

Note: ^*∗*^Calculations of *α* are based on complete case analysis. OQ-45 from sample 1: *n*_1_ = 588. OQ-45 from sample 3: *n*_3_ = 198. BSI from sample 2: *n*_2_ = 1282. BSI from sample 3: *n*_3_ = 237.

**Table 3:** Statistics of the OQ-45 and the BSI/SCL-90R based on samples from Germany.

	Functional population M (SD)	Dysfunctional population		Cut-off	Critical difference
		Outpatients M (SD)	Inpatients M (SD)		
OQ Total Score	46.2 (18.5) [20]	71.8 (21.9) [21]	79.0 (27.9) [18]^*∗*^	58.8 [18]	17.8 [18]
GSI	0.31 (0.23) [22]	1.08 (0.63) [23]	1.47 (0.68) [24]^†^	0.60 [24]^†^	0.2 [25]

Note: ^*∗*^Pooled M and SD from the intervention and control group at admission. ^†^Based on SCL-90R data.
